# Fear and Anesthesia

**Published:** 1914-12-15

**Authors:** Louis Schultz


### FEAR AND ANESTHESIA.

BY LOUIS SCHULTZ, D.D.S.

The most potent factor in favor of the use of nitrous oxid and oxygen as an analgesic is that it removes the anticipation, the fear on the part of the patient, and this occurs after the second or third inhalation usually. This fact alone is important enough to warrant the use of the method but it does more than that; it puts the patient's mind in a condition favorable to receive suggestions, so that the operator has this added advantage, aside from the fact that it actually does take away pain. There are some cases where a show of sympathy, combined with gentle manipulation, and the use of sharp instruments are all that is necessary. In others, we may be able to divert the mind of the patient from what we are doing by a discussion of some subject of great interest to the patient; in other cases still, local anesthesia may be the best method, but there remains a large number of cases, where I feel that I would not do my whole duty to my patient unless I removed his greatest objection of coming to a dental office, and that is the fear and the anticipation of the things that wait for him there. But I repeat, it does more than that; it actually removes the pain itself, it keeps our patients free from shock, it is not unpleasant to take, especially if, as De Ford suggests, the odor be disguised by a few drops of essence of orange placed in the inhaler; and, finally, it leaves no unpleasant after effects.

The very fact that it actually takes away the pain has been used as a point against it, as though the infliction of pain in the treatment of disease was a desirable thing. I know of but very few exceptions to the statement that pain caused by the treatment of a pathological condition is never beneficial. So well is this statement recognized by the medical profession the world over, that it is no longer a question, but an accepted fact. A surgeon operating without anesthesia would have nothing to operate; an accoucheur with-holding the anesthetic in cases of confinement would have nothing to confine; more than that, such an attempt would be likely to result in severe censure or worse. Very few operations are undertaken in surgery without some kind of an anesthetic, and those must be of very short duration, and in a field hard to anesthetize. There is no question about the use of an anesthetic in the extraction of teeth, and yet the pain inflicted in the preparation of some cavities is greater, and of longer duration, and I cannot help feeling, therefore, that the control of that pain, or better, its removal, is of even greater importance than the control of pain in the average extraction, especially since the analgesic state is all that is necessary to do it.

I am well aware of the fact that pain is one of nature's ways of protection. It is her method of help, as in burning, scalding, etc., so that we can save ourselves from serious injury or death; even a pulpitis is nature's way of inducing us to seek help and protection againts caries, but here it serves a useful purpose, and can not be likened to pain inflicted during treatment. Another view, to which I must take exception, is that the pain sense is necessary, and, in fact, is guiding us when approaching a live pulp in deep cavities, and helps us to keep from exposing it. I find that in every case treatment must be adapted to the conditions present, and if caries encroach upon the pulp to an extent that we fear its being infected, the pulp had better be destroyed in the average case, rather than to temporize with it, and later treat an abscess. If the tooth be not involved to such an extent, our knowledge of its anatomy should guide us while preparing the cavity. I am free to confess that I have opened into a few pulps quite unintentionally, before I used this method, and I am equally free to say, that since using this method, the accident has occurred no more frequently in my hands; but I find this difference, that I can excavate deep cavities better and easier and line them with some suitable non-conductor of thermal changes, and so save the pulp in those teeth, when I induce analgesia, than I could before I used this method. My experience, at least, contradicts the statement that to remove the sensibility in a deep cavity endangers the pulp.

Should this method then be used indiscriminately to control every form of pain in the dental office? I don't believe that an operator could be found who would so hold no more than we would advocate pulp extraction in every carious tooth, or the restoration of lost tooth substance by means of inlays in every case. I maintain, however, that there are a large number of cases where it is indicated. Whenever fear or pain would result in enough shock to warrant its use it should be employed, and that condition obtains in far more cases than where a good operation could not be done, or no operation could be made short of leaving a shattered nervous system from the excess of suffering. I hold it to be my duty to prevent shock whenever I can, and I, for one, can accomplish that with nitrous oxid and oxygen quicker and easier than in any other way; and I firmly believe it to be the duty of every operator to eliminate pain, no matter what method he employes. I think that probably I can accomplish more with this method by virtue of my long association with it, and I know that with its aid the patient loses what fear he may have, and so I prevent psychical shock and as soon as the state of analgesia is established the afferent nerve impulses are blocked sufficiently to prevent physical shock, due to the painful impressions incident to cavity preparation.—*Dental Review.*

